# Impact of nutrition counseling on nutrition status in patients with head and neck cancer undergoing radio- or radiochemotherapy: a systematic review

**DOI:** 10.1007/s00405-023-08375-1

**Published:** 2024-01-04

**Authors:** Jenny Zeidler, Sabine Kutschan, Jennifer Dörfler, Jens Büntzel, Jutta Huebner

**Affiliations:** 1 Klinik Für Innere Medizin II, Hämatologie Und Internistische Onkologie, Universitätsklinikum Jena, Am Klinikum 1, 07747 Jena, Germany; 2 Klinik Für HNO-Erkrankungen, Südharz-Klinikum Nordhausen, Dr.-Robert-Koch-Str. 39, 99734 Nordhausen, Germany

**Keywords:** Nutritional support, Nutritional therapy, Head and neck cancer, Malnutrition, Cachexia

## Abstract

**Purpose:**

In this systematic review, we included randomized controlled trials from 2004 to 2021 to determine the effect of individual dietary counseling for patients with head and neck cancer, specifically, nutritional outcome, morbidity, and quality of life (QOL), during and after chemo- and chemoradiotherapy.

**Methods:**

In October 2023, a systematic search was conducted searching five electronic databases (Embase, Cochrane, PsychInfo, CINAHL, and Medline) to find studies concerning the use and effectiveness of intensive nutritional care on head and neck cancer patients.

**Results:**

From all 2565 search results, 6 studies with 685 head and neck cancer patients were included in this systematic review. The patients were treated with radiotherapy or radiochemotherapy. The therapy concepts include intensive nutritional support from a dietician. Outcomes were nutritional status, body composition, quality of life, and adverse effect. All studies had low quality, high risk of bias, and reported heterogeneous results: some studies reported significant improved nutrition status, body composition and quality of life, while other studies did not find any changes concerning these endpoints.

**Conclusion:**

Due to the very heterogeneous results and methodical limitations of the included studies, a clear statement regarding the effectiveness of intensive nutritional therapy of head and neck cancer patients is not possible. Further well-planned studies are needed.

**Supplementary Information:**

The online version contains supplementary material available at 10.1007/s00405-023-08375-1.

## Introduction

Worldwide, head and neck squamous cell carcinomas (HNSCC) represent the sixth-most common type of cancer with approximately 900,000 cases annually [1]. Patients with head and neck cancer (HNC) have a high rate of malnutrition and cumulative weight loss of more than 10%, resulting in inadequate response to treatment, delayed wound healing, and occurrence of major postoperative complications that affect quality of life (QOL) and significantly lower survival [2–5].

An important factor is the weight loss and anorexia in untreated HNC patients before treatment, caused by symptoms such as dysphagia, odynophagia, and metabolic changes, resulting in 3–52% of naïve HNC patients being malnourished [6].

All types of treatment may increase the risk of malnutrition due to different side effects. With the onset of chemotherapy, (CT) radiotherapy (RT) or both (CRT), depletion in nutritional intake exacerbates due to the side effects such as xerostomia, mucositis, dysphagia, vomiting or nausea. Complications due to cutaneous endoscopic gastrostomy (PEG) tube placement and poor teeth status can further negatively affect the nutritional intake [7]. Due to the rigors of the treatment adequate nutritional intake and weight maintenance is extremely challenging [8, 9] and 20% of HNC withdraw from treatment [10]. As a result, morbidity and mortality of HNSCC patients increase [11].

After completion of treatment, HNC patients may experience late and long-term side effects, such as permanent saliva loss, taste dysfunction, pharyngoesophageal stenosis, which continue to make it difficult to achieve nutritional goals and maintain weight [12, 13].

Usually standardized nutritional counseling is offered to reduce weight loss and improve the outcome of the patient. However, this counseling is often not long term and the individuality of each patient is not sufficiently supported. In contrast, intensive and individualized nutritional support focuses on each patient’s body composition, nutritional intake and needs and clinical status during but also after active cancer treatment. Patients have regular consultations with oncology trained dieticians, check-ups and receive advice to reduce common side effects of cancer treatment. Given the current limitations on resources, such as trophologists, it becomes crucial to assess the availability of evidence supporting their necessity.

The aim of this review is to assess the clinical evidence on individualized nutritional counseling in head and neck cancer patients compared to standard care. Therefore, we conducted a systematic review evaluating patient-relevant endpoints like nutritional status, change in body weight, as well as protein and energy intake and quality of life.

## Methods

### Criteria for including and excluding studies in the review

Inclusion and exclusion criteria are listed in Table 1 based on a PICO model. Generally, all study types were included if they reported patient-relevant outcomes after treatment of adult head and neck cancer patients with intervention of intensive nutritional care by a dietician including the possible use of supplementary nutrition. Application of parenteral nutrition or the usage of PEG and PEJ tubes was not taken into consideration. Criteria for rejecting studies were primary prevention, gray literature, other publication type than primary investigation/report (e.g., comments, letters, abstracts), and study population with precancerous conditions. Additionally, studies were excluded if they reported no patient centered outcomes for example laboratory parameters. Language restrictions were made to English and German.

**Table 1:** Inclusion and exclusion criteria based on a PICO model

PICO	Inclusion criteria	Exclusion criteria
Patient	Head and neck cancer patients (all entities and stages)	Patients with precancerous conditions or carcinoma in situ Primary prevention Preclinical studies
Intervention	Every intervention based on nutritional support No restrictions regarding the length of the intervention, number of counseling sessions	
Comparison	All possible control groups (active control, placebo, standard/guideline/ usual care)	
Outcome	Mortality (overall survival) Morbidity (progression-/disease-free interval, tumor response) Patient-reported outcomes (PG-SGA score, quality of life) Nutritional deterioration Weight and body composition, energy and protein intake	
Others	Language: German and English Full publication	Gray literature (conference articles, abstracts, letters, ongoing Studies, unpublished literature, etc.) Full text not available in German or English

### Study selection

A systematic research was conducted using 3 databases (Medline (Ovid), EMBASE (Ovid), and Cochrane CENTRAL) in October 2023. For each of these databases, a complex search strategy was developed consisting of a combination of MeshTerms, keywords and text words in different spellings connected to head and neck cancer and nutritional support (eSupplement e1). The search string was highly sensitive, since it was not restricted by filters of study or publication type. After importing the search results into EndNote X9, all duplicates were removed and a title–abstract screening was carried out by two independent reviewers (JZ, JH). In case of disagreement, consensus was made by discussion. After that, all full texts were retrieved and screened again independently by both reviewers. When title and abstract did not have sufficient information for screening purposes, a full-text copy was retrieved as well. Additionally, bibliography lists of all retrieved articles were searched for relevant studies.

### Assessment of risk of bias and methodological quality

All characteristics were assessed by two independent reviewers (JZ, JD). In case of disagreement, a third reviewer was consulted (JH) and consensus was made by discussion.

The risk of bias in the included studies was analyzed with the Cochrane revised Risk of Bias Tool 2.0 [14]. Additional criteria concerning methodology were size of population, application of power analysis, adequacy of statistical tests (e.g., control of premises or multiple testing) and selective outcome reporting (report of all assessed outcomes with specification of statistical data as the p-value).

### Data extraction

Data extraction was performed by one reviewer (JZ) and controlled by two independent reviewers (JD, JH). As a template for data extraction, the evidence tables from the national Guideline on Complementary and Alternative Medicine in Oncological Patients of the German Guideline Program in Oncology [15] were used. Concerning systematic reviews, only data from primary literature meeting the inclusion criteria of the present work were extracted.

## Results

The systematic research revealed 2565 results. At first, duplicates were removed leaving 2127 studies. After screening title and abstract, 41 studies remained to complete review (see Consort diagram, eSupplement e2). One study was added by hand search. Finally, 6 RCTs were analyzed in this review. Detailed characterization of the included studies may be seen in Table 2.

**Table 2:** Characterization of the included studies:

Reference	*N*	Cancer site/ stage	Mean age/ male: female ratio	Intervention/duration	Control group	Body composition/Nutrition status at beginning	Endpoints	Outcome	Body composition after treatment	
									Intervention group	Control group
Isenring et al. [14]	*N = *60 IG (*n = *29) CG (*n = *31)	Head and neck area (88%) abdominal or rectal area (12%)	IG: 60.6/4.8 CG: 63.3/6.75	Regular and intensive nutrition counseling by a dietitian (start within the first 4 days of radiotherapy) weekly for the course of radiotherapy using predetermined standard nutrition protocol and the Medical Nutrition Therapy (Cancer/Radiation Oncology) protocol of the ADA, telephone reviews between nutrition counseling sessions, sample meal plans, recipe suggestions and hints to minimize the side effects Duration: 12 weeks after commencing radiotherapy	Outpatient dietitian, 2 dietetic consultations, education by the nurses Provision of the resource ‘Understanding Nutrition—a booklet from the Queensland Cancer Fund’, Oral nutrition supplement samples, Less nutrition assessment, No individualization of nutrition advice Less follow-up	Well-nourished *n = *39 (65%) Malnourished *n = *21 (35%), of which *n = *17 (28.3%) moderately nourished or suspected of being malnourished and *n = *4 (6.7%) severely malnourished	1. Bodyweight 2. Nutritional status 3. Global QoL 4. Body composition 5. Physical function	1. Significant more weight stable patients in the intervention group (*p* < 0.001) 2. Intervention group had significantly smaller deterioration in nutritional status (*p* = 0.020) 3. Significant smaller decrease and faster recovery in global QoL in the intervention group (*p* = 0.009) 4. Clinically, but not statistically significant differences in fat-free mass (*p* = 0.195) 5. Significantly faster recovery in physical function in the intervention group (*p* = 0.012)	Losing weight between the 4- and 8-week period regained weight and maintained body weight over 12 weeks (Mean change = -0.4 kg) weight stable subjects (24%), weight losing (22%) Mean gain of 0.5 kg FFM	Losing weight between the 4- and 8-week period greater deterioration in weight (Mean change = -4.7 kg) weight stable subjects (11%), weight losing (43%) Mean loss of 1.4 kg FFM
Ravasco et al. [17]	*N = *75 Group 1: *n = *25 Group 2: *n = *25 Control group *n = *25	Cancer of the base Tongue, nasopharynx, oropharynx, and larynx; Stage I/II (*n = *30) stage III/IV (*n = *45)	No differentiation between the groups 60/4.0	Group (G1): individually dietary support recognizing personal eating patterns and preferences, receiving prescription that identifying the type, amount, and frequency of feeding and specifying the caloric/protein level to attain, together with any restrictions and limited or increased individual dietary components Group 2 (G2): maintained usual diet plus supplements: 2 cans per day of liquid polymeric formulations, each can provides 20 g of protein and 200 kcal	Maintained intake ad libitum (CG)		1. Nutritional intake 2. Nutritional status (PG-SGA score) 3. Symptom-Induced Morbidity 4. Global QoL	1. Energy intakes significant increased in G1 (*p* = 0.002) and in G2 (*p* = 0.05); G1 > G2, *p* = 0.005) and decreased in CG (*p* < 0.01) at the end of RT, then at 3 months’ follow-up, G1 maintained their energy intake, G2 and CG decreased (*p* = 0.005), in regard to protein intake significant increase in G1 (*p* = 0.006), G2 (*p* = 0.001); group 1 < group 2 (*p* = 0.06) and decrease in CG (*p* < 0.01) at the end of RT, then at 3 months’ follow-up, G1 maintained protein intake, whereas G2 and CG decreased (*p* < 0.05) 2. Significant statistical differences between intervention groups, regarding nutritional decline both at the end RT and at 3 months (*p* < 0.002), significant statistical differences between intervention groups, regarding maintenance/improvement of nutritional status at the end RT and at 3 months (*p* < 0.001) 3. No significantly between-group difference in morbidity (*p* < 0.08), although a trend for reduced symptoms was found in G1 versus G2 and CG (*p* < 0.07) at end of RT, at 3 months’ follow-up significant reduction in the incidence and severity of grade 1 + 2 anorexia, nausea/vomiting, xerostomia, and dysgeusia G1 vs G2 vs CG (*p* < 0.0001), G1 > G2 and CG (*p* < 0.07) 4. Global QoL function scores significantly improved at the end of RT in G1 (*p* < 0.003) and G2 (*p* < 0.009) and worsened in CG, at 3 months’ follow-up all G1 patients maintained or improved their overall QOL, G2 patients maintained or experienced a decline in their overall QOL (*p* < 0.03), CG function scores further deteriorated (*p* < 0.004)		
Roussel et al. [18]	*N = *87 IG (*n = *43) CG (*n = *44)	Cancer of oropharynx (*n = *40), nasopharynx (*n = *2), hypopharynx (*n = *11), larynx (*n = *19), sinus (*n = *2), oral cavity (*n = *9), unknown (*n = *4) stage x (*n = *5) stage I (*n = *1) stage II (*n = *8) stage III (*n = *14) stage IV (*n = *58) stage V (*n = *1)	IG: 62/3.3 CG: 59/5.3	Standard care as control group and additionally 6 individualized meetings with the dietitian at home (2 during the radiotherapy, 4 at the end of the radiotherapy and then 2 months later)	2 outpatient consultations with a dietitian during radiotherapy using ESPEN guidelines on enteral nutrition: non-surgical oncology and American Dietetic Association protocols: The clinical guide to oncology nutrition, evaluating diet history, assessment of the patient’s qualitative and quantitative current intake, patient’s weight history, energy, and protein requirements, nutritional adjustments	Mean weight: IG: 72.0 kg CG: 69.0 kg BMI (kg/m^2^) IG: 24.4 CG: 24.0	1. EORTC QLQ-C30 version 3 2. EORTC QLQ H&N35 3. EQ-5D-3L 4. EQ VAS 5. Weight and BMI change 6. Energy and protein intakes	1. Significant deterioration in the item sleep in CG (*p* = 0.04) 2. At 3 months: more cough symptoms in CG (*p* = 0.04); mean change from baseline to 3 months: significant deterioration for item “speech” for CG, improvement in IG (*p* = 0.02) 3. No significant difference between groups 4. No significant difference between groups 5. Insignificant BMI loss for IG 1.2 kg/m^2^ (1.7) and for CG 1.5 kg/m^2^ (2.5) (*p* = 0.63) 6. No significant differences in energy intake (*p* = 0.41) and protein intake (*p* = 0.50) at 1 month after radiotherapy or after 3 months (*p* = 0.07 and *p* = 0.79, respectively), no significant energy and protein change from 1 to 3 months after radiotherapy (*p* = 0.22 and *p* = 0.99, respectively)	Mean weight: 68.4 kg Mean BMI (kg/m^2^): 22.8	Mean weight: 64.6 kg Mean BMI (kg/m^2^): 22.5
Britton et al. [19]	*N = *307 IG (*n = *151) CG (*n = *156)	Cancer of (nasopharynx (*n = *23), oropharynx (*n = *171), oral cavity (*n = *66), larynx (*n = *29), hypopharynx (*n = *11), unknown primary (*n = *7) stage I (*n = *12) stage II (*n = *39) stage III (*n = *57) stage IV (*n = *199)	IG: 58/3.8 CG: 58/4.2	Oncology trained dietitians delivered EAT during usual consultations, weekly exposure while the patient was receiving RT and then every 2 weeks EAT (used CBT strategies and motivational interviewing) 1. principle: MI interactional style of a collaborative, empathic conversation 2. principle: having a concrete plan 3. principle: recorded externally 4. principle: importance, achievable and is being monitored “Eat to LIVE” conversation (after 5 weeks of RT) 1) Why are you having radiotherapy? 2) I wonder if I can tell you something about malnutrition during treatment? 3) I’m puzzled by the difference between what you want and what you are currently doing with your nutrition 4) What’s the next step?	Treatment as usual, making no changes to any part of their clinical care	Mean Nutritional status (PG-SGA) IG: 5 CG: 5	1.PG-SGA score 2. SGA category, weight loss, > 10% weight loss 3. PHQ-9 depression score 4. RT interruption 5. Unplanned admissions 6. Mean length of stay, total days 7. Total HRQOL score (QLQ-C30 score)	1. IG significantly lower (better) scores compared to control group at the end of RT (*p* = 0.03) 2. IG significantly more likely to be in SGA category A: (*p* < 0.01), Significantly more control participants experienced weight loss (*p* = 0.03) and also more than 10% weight loss (*p* = 0.03) 3. IG had significantly lower depression scores CG (*p* = 0.04) 4. CG had more treatment interruptions (*p* = 0.04) 5. No significant differences between groups 6. IG had significantly better overall QOL (*p* < 0.01)	Mean Nutritional status (PG-SGA): 14.71	Mean Nutritional status (PG-SGA): 16.24
Orell et al. [20]	*N = *58 IG (*n = *26) CG (*n = *32)	Advanced (Stage III + IV) squamous cell carcinoma of the oral cavity, oropharynx, hypopharynx, nasopharynx, or larynx	IG: 57/4.2 CG: 61/3.6	Protocol counseling given by a dietician at baseline, on the 2nd and 4th weeks of treatment and at the end of chemoradiotherapy. During chemoradiotherapy: protocol assessment of nutritional intake and fine-tuning nutrition plan or nutrition for tube feeding according to treatment side effects	Patients received protocoled nutritional counseling, which included dietary prescription (i.e., tube feeding, product, and volume) and counseling for energy- and protein-dense texture-modified diet via a dietary booklet booklet that included meal plans and recipes for energy- and protein-dense texture-modified meals for oral intake designed for the current study	BMI, kg/m^2^, n (%) < 20: IG *n = *3 (12) CG: *n = *6 (19) 20–25: IG: *n = *9 (35) CG: *n = *15 (47) > 25: IG: *n = *14 (54) CG: *n = *11 (34) Malnourished (PG-SGA BC), n (%) IG: *n = *7 (27) CG: *n = *14 (44) Critical pre-treatment weight loss: IG = 23% (*n = *6/26) CG = 25% (*n = *8/32) BMI at baseline: IG: 24.8 CG: 23.1 PEG, n (%) IG: *n = *20 (78) CG: *n = *31 (97)	1. Nutritional status (PG-SGA) 2. Weight loss 3. Handgrip strength (HGS) 4. Body composition 5. Survival	1. No significant differences between the groups on nutritional status (PG-SGA score) but median PG-SGA score for all patients increased significantly during treatment (*p* < 0.001) 2. No significant differences on weight loss (*p* = 0.704) 3. Median HGS in IG was not significantly different between the study groups (*p* = 0.803) 4. No significant differences between groups weight, kg (*p* = 0.690); BMI, kg/m2 (*p* = 0.656); FFMI (fat-free mass index), kg/m2 (*p* = 0.741), FFM (fat-free mass), kg (*p* = 0.684); FM (fat mass) kg (*p* = 0.332) 5. No significant differences between groups but lower baseline HGS (*p* = 0.05) and malnutrition (*p* = 0.014) were associated with worse DFS	BMI, kg/m^2^: 23.3	BMI, kg/m^2^: 22.4
Löser et al. [21]	*N = *61 IG (*n = *33) CG(*n = *28)	Cancer of the oropharynx (*n = *38), oral cavity (*n = *10), hypopharynx (*n = *4), larynx (*n = *5), other (*n = *4) UICC stage I (*n = *19) II (*n = *11) III (*n = *11) IV (*n = *20)	IG: no data/ 3.1 CG: no data/ 2.1	Individual nutritional counseling based on an assessment of the diet diary, bioelectrical impedance analysis (BIA), blood count and the clinical condition of the patient regularly reassessment of the nutritional status by means of BIA and continuous nutritional counseling every two weeks lasting 30 min	Regular medical check-ups with a physician	BMI, kg/m^2^, at baseline: IG: 24.4 ± 5 CG: 24.4 ± 4.2	1.Phase Angle (PA) 2.Weight loss and deterioration of FFMI 3. BMI 4.Caloric deficit 5.Laboratory parameters 6.Therapy-related side effects 7. Survival rate	1. No significant differences of the PA between the groups (*p* = 0.91) 2. Insignificant weight loss and deterioration of FFMI between the groups (*p* = 0.82) 3. No relevant differences between both arms with respect to changes in BMI (*p* = 0.46) 4. The measurement of the caloric deficit at the start of the intervention showed no significant difference between the groups (*p* = 0.772). Within the overall patient population, the calorie deficit rose significant between the start and the end of the intervention (*p* = 0.001) 5. Only significant difference in total protein count (*p* = 0.012) 6. Therapy-related side effects showed no significant differences 7. Insignificant overall survival rate between the groups (log-rank *p* = 0.79). Patients with a FFMI of < 15 (female) and < 17 (male) kg/m^2^ at the end of the intervention presented with a shorter overall survival (log-rank *p* = 0.008). Patients with albumin levels > 24.5 g/L presented with a longer overall survival (log-rank *p* = 0.016)		

*IG* intervention group, *CG* control group

### Characteristics and description of included studies

Concerning all relevant studies, 685 patients were included and 634 of them were analyzed, due to 51 drop-outs. The mean age of patients (reported in 5 studies) ranged from 58 to 63 years with a range of age (reported in 4 studies) from 20 to 89 years. Information about the gender of the included patients could be obtained for 568 of the 685 patients. Out of these 568 participants, 118 (20,8%) were female and 450 (79,2%) were male.

The intervention group in the study by Isenring et al. [16] received regular and intensive nutritional counseling by a dietitian within the first four days after the start of radiotherapy and weekly throughout the course of radiotherapy (approximately 6 weeks) and fortnightly during the remainder of the study period using a predetermined standard nutritional protocol and the ADA Protocol for Medical Nutrition Therapy (Cancer/Radiation Oncology). Additionally, telephone discussions have been conducted between nutrition counseling sessions, along with providing sample meal plans, recipe suggestions, and tips on minimizing side effects and if necessary, a weekly supply of oral nutritional supplements. The study began at the start of radiotherapy and lasted 12 weeks.

In the study of Ravasco et al. [17], intervention group 1 received individualized dietary counseling that took into account personal eating habits and preferences. The prescribed regimen detailed the precise type, quantity, and frequency of feeding, while also specifying the caloric and protein levels to achieve. Furthermore, any dietary limitations or modifications pertaining to specific components were explicitly outlined, such as restricted or augmented intake of individual dietary components. Intervention group 2 received two doses a day of ready-to-use, high-protein, energy-dense liquid polymeric formulations designed to serve as a supplement to the patient's usual diet. Each 200 mL can provide 20 g of protein and 200 kcal. The study began at the start of radiotherapy and lasted 12 weeks.

In addition to usual care, the intervention group of Roussel et al. [18] received 6 individualized meetings with a dietitian. Two consultations during radiotherapy and 4 after the end of radiotherapy. The study began at the start of radiotherapy and lasted 12 weeks.

In the study by Britton et al. [19], the intervention group received counseling from oncology dietitians weekly during radiochemotherapy and every 2 weeks thereafter, based on motivational interviewing and cognitive behavioral therapy. The study began at the start of radiotherapy and ended 12 weeks after the end of radiotherapy.

In the study by Orell et al. [20], the intervention group received nutritional counseling by a dietitian at baseline, week 2 and 4 of treatment, and at the end of chemoradiotherapy. During chemoradiotherapy, the intervention group received protocol-based assessment of dietary intake and a detailed nutrition plan depending on the side effects of treatment. The study lasted from the beginning chemoradiotherapy with a minimum follow-up time of 40 weeks or until death.

In the prospective study by Löser et al. [21], the intervention group received individual nutritional counseling based on an assessment of the diet diary, bioelectrical impedance analysis (BIA), blood count, and the clinical condition of the patient, including the presence of a feeding tube. Subsequently individual dietary recommendations were given by the dietitians. In addition, in the intervention group, the nutritional status was regularly reassessed by means of BIA and continuous nutritional counseling was provided every two weeks. Nutritional consultations in the intervention group lasted around 30 min. In contrast, the patients in the control group did not receive individual nutritional counseling, but instead had regular medical check-ups with a physician.

### Excluded studies

A list of the studies excluded after full-text screening and the reason for exclusion are presented in Table 3.

**Table 3:** Studies excluded after full-text screening

Author	Year	Title	Type	Reason for exclusion
Capozzi et al. [24]	2016	Patient-reported outcomes, body composition, and nutrition status in patients with head and neck cancer: results from an exploratory randomized controlled exercise trial	RCT	Due to the additional physical exercise program during the intervention the outcome cannot be attributed to the nutritional counseling alone
Qiu et al. [25]	2020	Effect of whole-course nutrition management on patients with esophageal cancer undergoing concurrent chemoradiotherapy: a randomized control trial	RCT	Different cancer type

### Risk of bias in included studies

The methodical quality was assessed with RoB2 tool, and the results are presented in Table 4. All of the included studies have a high risk of bias.

**Table 4:**
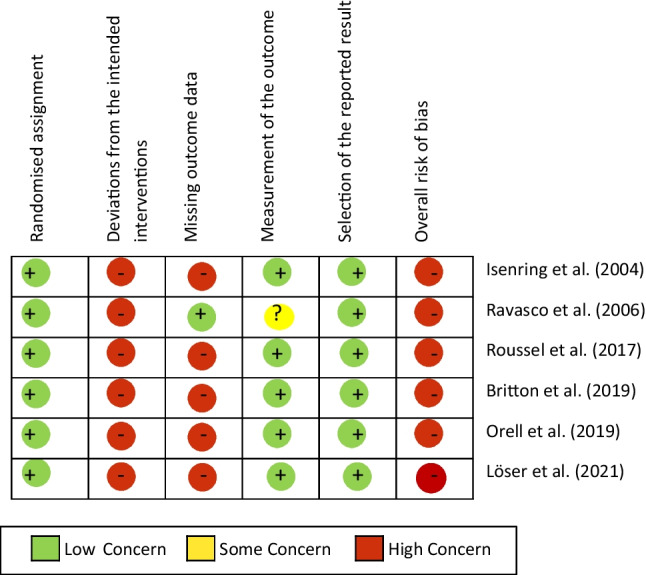
Risk of bias in the included randomized controlled studies according to the Cochrane risk of bias tool

### Efficacy of (intensive/individual) nutritional support

#### Survival and disease progression

Overall survival was analyzed in two RCTs [20, 21]. In the study of Orell et al. [20], the 5-year overall survival (OS) for HNC patients showed no difference between the intervention and control group (*p* = 0.81). There were no significant between-group differences for disease-specific survival (DSS) (*p* = 0.562), as well as disease-free survival (DFS) (*p* = 0.939).

The study of Löser et al. [21] showed insignificant overall survival rate between the groups (log-rank p = 0.79). Patients with a FFMI of < 15 (female) and < 17 (male) kg/m^2^ at the end of the intervention presented with a significant shorter overall survival (log-rank *p* = 0.008). Patients with albumin levels > 24.5 g/L presented with a significant longer overall survival (log-rank *p* = 0.016).

#### Feasibility and adherence

Out of the included 685 patients, which were included in the 6 studies, 51 drop-outs occurred (7.4%), mainly due to side effects of radiochemotherapy. From the 51 drop-outs, 24 (47%) were part of the intervention group and 27 (53%) of the control group. [16–21].

#### Nutritional status and calorie deficit

In four studies, nutritional status was assessed using the Scored Patient-Generated Subjective Global Assessment score (PG-SGA). This is an interdisciplinary patient assessment tool for oncology patients.

Isenring et al. [16] reported a mean PG-SGA score of 6.4 in the intervention group and 5.3 in the control group at baseline and 4.8 and 8.4, respectively, at 12 weeks (*p* = 0.02). Thus, patients in the intervention group had less deterioration in nutritional status as indicated by a lower PG-SGA score compared to the control group.

In the study by Ravasco et al. [17], 8 out of 16 malnourished patients receiving dietary counseling improved their PG-SGA score after 3 months. In intervention group 2 (supplements) and the control group, none improved their nutritional status. No statistical comparisons between the beginning and the end of the intervention were presented.

In Britton et al. [19], the intervention group showed significantly less deterioration in nutritional status than the control group (*p* = 0.03). No statistical comparisons between the beginning and the end of the intervention were presented.

Orell et al. [20] reported no significant difference in nutritional status between the intervention and control group. The statistical value was not provided. Importantly, Orell et al. [20] did not compare the intervention to a control group with no nutrition support at all, but with an individualized on-demand nutritional counseling.

The study of Löser et al. [21] analyzed the calorie deficit, calculated by the difference between the actual calorie intake and the calculated, necessary calorie intake to maintain the current body weight. The measurement of the caloric deficit at the start of the intervention showed no significant difference between the groups (*p* = 0.772). Within the overall patient population, the calorie deficit rose significant between the start and the end of the intervention (*p* = 0.001).

### Change in body weight

The studies indicated a trend of comparatively lower weight loss in the intervention groups as opposed to the control groups.

In Isenring et al. [16], the intervention group maintained their body weight (mean change = 0.4 kg) during the 12 weeks compared to a significantly larger loss of weight loss in the control group (mean change = 4.7 kg) (*p* = 0.001). More subjects in the intervention group were weight stable and more subjects in the control group were weight losing (*p* = 0.016).

The study of Ravasco et al. [17] reported, over a period of 3 months, a loss of weight in only 20% of participants receiving dietary counseling, 76% in participants taking supplements, and 96% in the control group. Only participants in the group receiving dietary counseling were able to gain weight with a mean of 4 kg (range: 2–6 kg).

In the study by Roussel et al. [18], there were no significant differences in body weight or reduction of BMI between the intervention and control groups (*p* = 0.475) from baseline to 3 months after radiotherapy. The intervention group had a mean decrease of 3.6 kg and the control group one of 4.4 kg, resulting in BMI loss of 1.2 kg/m^2^ and 1.5 kg/m^2^, respectively (*p* = 0.63). There was also no significant weight or BMI loss between groups from the start of the study to 1 month after radiotherapy.

The study by Britton et al. [19] showed weight loss in almost all patients, but significantly greater weight loss in the control group than in the intervention group (*p* = 0.03). In addition, more participants in the control group had weight loss greater than 10%. (*p* = 0.03).

In another study by Orell et al. [20], a total of 71% of all patients had lost more than 5% weight, with a mean weight loss of 7.7% at the end of chemoradiotherapy. Critical weight loss occurred in both groups: 77% in the intensive nutrition support group and 67% in the on-demand nutrition support group (*p* = 0.704). In addition, the prevalence in both groups of overweight patients decreased from 43 to 26% (*p* = 0.921) and underweight patients increased from 16 to 28% (*p* = 0.012). There were no significant differences between groups (intervention 41%, control 47%) in patients who remained within the normal weight range.

In the study by Löser et al. [21], weight loss and worsening of FFMI were found in both groups, with no significant difference between the control group and the intervention group (*p* = 0.82). Patients with severe trismus (grade 3/4) tended to have a significant lower FFMI at the beginning and also at the end of the intervention, respectively (*p* = 0.004, *p* = 0.011). In terms of changes in the BMI, no significant changes were found between the groups (*p* = 0.46). However, the patients with a BMI < 22 kg/m^2^ showed less weight loss than all other subgroups tested (95%-CI: 0.33–2.95, *p* = 0.015).

### Incidence and severity of adverse effect due to radio- or radiochemotherapy

Four RCT monitored adverse effects due to radio- or radiochemotherapy.

The study by Ravasco et al. [17] reported the incidence and severity of nausea/vomiting, xerostomia, dysgeusia, and/or dysphagia/odynophagia. RT-induced toxicity occurred in more than 90% of patients at the end of radiotherapy. Although there was no significant difference between groups (*p* < 0.08), a trend toward decreased symptoms was noted in intervention group 1 compared with intervention groups 2 and control group (*p* < 0.07). After 3 months, the incidence and severity of grade 1 and 2 anorexia, nausea/vomiting, xerostomia, and dysgeusia had decreased in all three groups, although there were significant differences. In intervention group 1, 90% of patients improved, in intervention group 2 67% improved, and the control group 51% improved resp. (*p* < 0.0001). There was no significant reduction in the incidence and severity of grade 1 + 2 dysphagia/odynophagia between groups (*p* < 0.09).

In the RCT by Orell et al., [20] there was no significant difference in the incidence of severe mucositis between the intervention and control group (*p* = 0.161). More severe mucositis occurred in patients with a weight loss of > 10% than in patients with a weight loss ≤ 10% (*p* = 0.692). Nausea occurred significantly more often in patients with a weight loss > 10% than in patients with weight loss ≤ 10% (*p* = 0.01).

Britton et al. [19] reported significant differences in nausea/vomiting and loss of appetite. (*p* < 0.01, *p* = 0.02). The incidence of fatigue, pain, dyspnea, insomnia, constipation, and diarrhea showed no significant differences between groups.

The prospective study by Löser et al. [21] showed no significant difference in therapy-related side effects between the groups. Only patients with more severe trismus (grade 3/4) tended to have a lower FFMI at the beginning and also at the end of the intervention, respectively (*p* = 0.004, *p* = 0.011).

### Energy and protein intake

Three studies monitored energy and protein intake.

In the study by Ravasco et al. [17], patients' energy and protein intakes were measured in comparison to estimated energy requirements (EER) and median reference values for protein. At baseline, there were no significant differences between the three groups. At the end of RT, energy intake showed a net increase of 521 kcal/day in intervention group 1 (*p* = 0.002) and of 322 kcal/ in intervention group 2 (*p* = 0.05); intervention group 1 > intervention group 2, (*p* = 0.005). Energy intake decreased by 400 kcal/day in the control group (*p* < 0.01). After 3 months, only intervention group 1 was able to follow the dietary recommendations and maintain their energy intake. Patients in intervention group 2 and the control group significantly decreased their energy intake to or below baseline (*p* = 0.005). As for protein intake, there was a net increase of 26 g/day in intervention group 1 (*p* = 0.006) and 35 g/day in intervention group 2 (*p* = 0.001); intervention group 1 < intervention group 2, (*p* = 0.06). In both intervention groups, the increase was significantly higher in patients with stage I/II disease, *p* = 0.05. Protein intake decreased in the control group (*p* < 0.01). At the 3-month follow-up, patients in intervention group 1 adhered to the dietary recommendations as during RT and maintained their protein intake, whereas patients in intervention group 2 and the control group decreased their protein intakes (*p* < 0.005) either to baseline or below baseline.

In the randomized controlled trial by Roussel et al. [18], no significant differences in energy and protein intake were found between groups, either 1 month after radiotherapy (*p* = 0.41 and *p* = 0.50, respectively) or 3 months after the end of radiotherapy (*p* = 0.07 and *p* = 0.79, respectively).

The RCT of Orell et al. [20] found no significant differences between the two study groups regarding the energy and protein intake. At the end of treatment, the median of the total energy intake was 82% of the estimated requirement, and the median protein intake was 72% of the estimated requirement. Specifically, the median energy intake in the intervention group (IG) was 27.5 kcal/kg (2,000 kcal/day) and 29.5 kcal/kg (1,950 kcal/day) in the control group (CG) (*p* = 0.24, NS). With regard to energy intake, 26% of all patients achieved more than 90% of the estimated energy requirement, 19% in the IG group and 31% in the CG group (IG vs. CG, *p* = 0.06). In addition, 12% of all patients achieved > 90% of the of the estimated protein requirement: three patients in the IG and four in the CG group, (*p* = 0.243).

### Laboratory parameters

In their conducted study, Löser et al. [21] exclusively investigated a comprehensive array of laboratory parameters, encompassing blood cell counts, inflammation markers, protein levels, metabolic markers, renal function, B-vitamins, and iron. Modifications in these parameters were predominantly observed in patients receiving concurrent chemotherapy in both the control and intervention groups. The sole significant contrast between these two groups was noted in the total protein levels of patients undergoing simultaneous chemotherapy. In the intervention group, the median total protein count was 69 g/L; whereas in the control group, it was 61 g/L (*p* = 0.012).

### Phase angle measurement

The study conducted by Löser et al. [21] exclusively analyzed the phase angle (PA), which is the tan value of the ratio of reactance versus electric resistance and depends on cell membrane integrity and body cell mass. There were no significant differences between the two study arms (*p* = 0.91). Furthermore, there were no relevant differences between the end of the intervention and the first follow-up regarding the PA (*p* = 0.59).

### Quality of life

Quality of life (QoL) was assessed in four randomized controlled trials using different tools.

In the study by Isenring et al. [16], QoL, assessed using the EORTC QLQ-C30 score, was lowest in both groups at 4 weeks. A stronger positive trend was seen in the intervention group during the 8-week period. Overall, the intervention group had a significantly lower decline and faster recovery in global quality of life compared to the control group (*p* = 0.009).

Ravasco et al. [17] assessed QoL at baseline, at the end of RT, and after 3 months using the EORTC QLQ-C30 score. In intervention group 1, all QoL function scores improved significantly after the end of RT (*p* < 0.003). There was a linear positive relationship between QoL score and nutritional status (*p* < 0.05) and energy and protein intake (r < 0.83; *p* < 0.001). All functional scores improved in intervention group 2 (*p* < 0.009), but they were associated only with the increase in protein intake. (*p* < 0.58; *p* < 0.05). In terms of symptom scales and individual items, all three groups worsened during RT, with the control group showing the greatest deterioration, which was associated with worsening food intake (*p* < 0.0001) as well as nutritional status (*p* < 0.002). In addition, sleep disturbance, appetite, and dyspnea worsened by the end of RT (*p* < 0.002). At the 3-month follow-up, all patients in intervention group 1 had either maintained or improved their overall quality of life, associated with a positive trend toward maintenance and improvement in nutritional status (*p* < 0.008) and adequate food intake (*p* < 0.01). Symptom scales and individual items were even significantly better compared to baseline scores. (*p* < 0.002). In intervention group 2, overall quality of life declined (*p* < 0.03) and scores for physical, role, emotional, and social functioning worsened (*p* < 0.07), only pain improved marginally (*p* < 0.06). These significant deteriorations were associated with inadequate dietary intake (*p* < 0.003) as well as depleted nutritional status (*p* < 0.002). Control group function scales, symptom scales, and individual scores remained poor compared with the end of RT and worsened with baseline scores. The significant deterioration, with the exception of pain, was related to inadequate dietary intake (*p* < 0.001) and deficient nutritional status (*p* < 0.002).

Roussel et al. [18] used the physical component summary of the EORTC H&N35 module to assess patients' quality of life (QoL) and functioning. From baseline to 3 months, there was a significant difference between groups in the item 'speech', which showed worsening in the control group and improvement in the intervention group (*p* = 0.02). After 3 months, the item 'cough' showed significant differences; the control group had more cough symptoms than the INC group (*p* = 0.04). The EQ-5D-3L and EQ VAS instruments showed no significant changes between groups at baseline, 1 and 3 months after radiotherapy.

Britton et al. [19] used the QLQ-C30 summary scale to assess the QL score (*p* < 0.01). Patients in the intervention group had a significantly better overall QoL score compared with the control group. This may be associated with significant differences in nausea and vomiting (*p* < 0.01), loss of appetite (*p* = 0.02), and physical (*p* = 0.01) and cognitive (*p* < 0.01) functioning.

### Physical function

Physical function was measured in 2 RCTs.

Isenring et al. [16] reported a significant difference in physical function between groups during the 12-week period (*p* = 0.012). Patients in the intervention group improved their physical function, whereas the control group remained impaired in physical function.

Orell et al. [20] measured patients' handgrip strength (HGS) using a dynamometer. The result showed no significant differences between the intensive nutritional care group and the on-demand care group.

## Discussion

This systematic review evaluated the impact of nutritional counseling on patients diagnosed with head and neck cancer who are undergoing radiotherapy. Four studies assessed nutritional status and intake using the PG-SGA score. Three of these studies indicated a significant improvement in nutritional status among patients who received nutritional interventions. In five studies measuring body composition, only two showed a smaller deterioration in weight in the intervention group. QoL was assessed in the majority of studies using the EORTC QLQ-C30 instrument. Most of these studies reported a significantly better QoL in the intervention group, but one study did not find a significant difference. The studies also identified various side effects of radio or radiochemotherapy. Some of these side effects, such as coughing, loss of appetite, and nausea and vomiting, had a lower incidence and severity in the intervention group. Looking closer at the seemingly heterogeneous results, there are several observations which could explain the heterogeneity and help to derive recommendations.

First of all, the studies by Isenring et al. [16], Ravasco et al. [17], and Britton et al. [19], found a positive effect, when nutritional counseling takes place weekly during radiation. In the three studies by Roussel et al. [18], Orell et al. [20] and Löser et al. [21], which found no effect of intensive nutritional counseling, nutritional counseling occurs only a total of 2 or 3 times or fortnightly, respectively, during radiation. This shows that the frequency of nutritional counseling during therapy significantly influences the outcome of the study.

Second, a potential limitation of all studies is that there was no true control group, as participants who received usual care still received an intervention. Yet, it would not be ethical to form a control group without any nutritional counseling. Every cancer patient should receive nutritional counseling because of the rigorous therapy, as this has a significant impact on survival [22].

Third, usual care, received by the control group, is not comparable, as each institution worldwide uses different nutritional guidelines. Moreover, the exact intensity of nutritional counseling of the control groups is not described in sufficient detail in each study. It is also important to investigate, who was advised by the dieticians, the patient himself or additionally the relatives, who might prepare the meals. Additionally, it should be noted that in the study by Ravasco et al. [17] the intake of supplements is energetically underbalanced.

Fourth, the duration of intervention, beginning with the start of the cancer treatment, and including follow-up appointments, varies from 3 months up to 63 months. It may not be possible to achieve significant results if the duration of the intervention is very short. In addition, it should be investigated the extent to which the start of the intervention in relation to the start of cancer therapy may have an impact on outcome. For example, a retrospective study on a nutritional intervention in esophageal cancer patients provided evidence that survival may improve if the intervention starts before chemoradiotherapy, suggesting the importance of early assessment and initiation of nutritional support [23].

There are some limitations to this systematic review. Due to the character of the intervention, blinding was not possible. Therefore, in this systematic review all studies were classified as highly biased. Thus, at least regarding quality of life, we may not derive, whether an improvement is a specific effect from nutrition intervention or an unspecific one by attention. Furthermore, only in the study by Ravasco et al. [17] were all participants able to complete the trial. In the other studies, dropout rates varied widely. Major reasons for discontinuation were: patient death, serious adverse events, withdrawal of consent, and failure to appear for follow-up. No study has documented the satisfaction of nutritional counseling. Also, the studies are very heterogeneous in terms of the nutritional status of the study population at baseline, ranging from severely malnourished to obese. Conclusions are difficult to ascertain from the available data, due to a high level of bias in most studies, short intervention time a small number of patients and high dropout rates, further increasing especially allocation, and performance bias.

### Limitations of this work

Some limitations of this systematic review must be mentioned. For once, due to the heterogeneity of the included RCTs no meta-analysis could be conducted, and no moderators of the effects caused by intensive nutritional support could be determined. Furthermore, only studies published in English or German were included in this review.

## Conclusion

Overall, even the most recent randomized controlled trials of individualized nutritional support in patients with head and neck cancer lack evidence of significant outcomes including nutritional status, quality of life, and side effects. More robust and consistent clinical evidence that includes comparable patient groups with comparable methodology, more detailed nutrition protocols, and consistent outcomes are needed to form a final judgment about the efficiency of individualized nutritional support in head and neck cancer patients.

### Supplementary Information

Below is the link to the electronic supplementary material.

Supplementary file1 (DOCX 17 KB)

Supplementary file2 (DOCX 27 KB)

## Data Availability

All data generated or analyzed during this study are included in the article.
