# Supplementary material for: Impact of nutrition counseling on nutrition status in patients with head and neck cancer undergoing radio- or radiochemotherapy: a systematic review
Source: Eur Arch Otorhinolaryngol. 2024 Jan 4;281(5):2195–209. doi: 10.1007/s00405-023-08375-1 (PMC11023997; doi:10.1007/s00405-023-08375-1)
Supplement: Supplementary file 1 — Supplementary file1 (DOCX 17 KB) [file 405_2023_8375_MOESM1_ESM.docx]

eSupplement e1

Vocabulary list utilized for systematic search

Vocabulary in OVID

| 1 | exp "Head and Neck Neoplasms"/ |
| --- | --- |
| 2 | exp laryngectomy/ |
| 3 | exp neck dissection/ |
| 4 | exp pharyngectomy/ |
| 5 | ((head adj2 neck) and (neoplasm$ or cancer$ or tumo?r$ or malignan$ or oncolog$ or carcinom$ or $sarcom$)).tw. |
| 6 | ((Mouth$ or Oral$ or gingival$ or neukoplakia$ or lip$ or palatal$ or tongue$ or nose$ or sinus$ or (salivary adj2 gland$) or Nasopharyn$ or oropharynx$ or Throat$ or oropharyng$ or hypopharynx$ or laryn$ or trachea$ or glotti$ or esophageal$ or facial$ or eyelid$ or Otorhinolary$ or ear$ or nose$ or pharyng$ or parathyroid$ or thyroid$ or papillary$) adj3 (neoplasm$ or cancer$ or tumo?r$ or malignan$ or oncolog$ or carcinom$ or $sarcom$ or metastas$ or nodule$)).tw. |
| 7 | (((head adj2 neck) and surg*) or ((head adj2 neck) and operat*) or (neck and dissect*) or laryngectom$ or pharyngectom$ or HNSCC or SCCHN).ti,ab. |
| 8 | (1 or 2 or 3 or 4) and (5 or 6 or 7) |
| 9 | exp diet therapy/ |
| 10 | (medic$ nutrition$ therap$ or cachexia$ or malnutrition$ or sarcopenia$ or clinical$ nutrition$ therap$ or medic$ diet$ therap$ or therapeutic$ diet$ or nutritional$ care$ or nutrition$ assessment$ or functional$ food$ or immunological$ diet or immunological$ nutrition$ or nutraceutical$).tw. |
| 11 | 9 or 10 |
| 12 | 8 and 11 |
| 13 | limit 12 to english or limit 12 to german |
| 14 | (13 and humans/) or (13 not animals/) |
| 15 | (((comprehensive* or integrative or systematic*) adj3 (bibliographic* or review* or literature)) or (meta-analy* or metaanaly* or "research synthesis" or ((information or data) adj3 synthesis) or (data adj2 extract*))).ti,ab. or (cinahl or (cochrane adj3 trial*) or embase or medline or psyclit or (psycinfo not "psycinfo database") or pubmed or scopus or "sociological abstracts" or "web of science" or central).ab. or ("cochrane database of systematic reviews" or evidence report technology assessment or evidence report technology assessment summary).jn. or Evidence Report: Technology Assessment*.jn. or (network adj1 analy*).ti,ab. or (((review adj5 (rationale or evidence)).ti,ab. and review.pt.) or meta-analysis as topic/ or Meta-Analysis.pt.) |
| 16 | (Randomi?ed controlled trial? or controlled clinical trial?).pt. or randomi?ed.ti,ab. or placebo.ti,ab. or drug therapy.sh. or randomly.ti,ab. or trial?.ti,ab. or group?.ti,ab. |
| 17 | 14 and (16 or 15) |
| 18 | 14 not 17 |

| 1 | mh Head and Neck Neoplasms |
| --- | --- |
| 2 | mh laryngectomy |
| 3 | mh neck dissection |
| 4 | mh pharyngectomy |
| 5 | ((Head NEAR neck) AND (neoplasm* or cancer? or tum*r? or malignan* or oncolog* or carcinom* or lymphoma? or sarcoma?)) |
| 6 | ((Mouth$ or Oral$ or gingival$ or neukoplakia$ or lip$ or palatal$ or tongue$ or nose$ or sinus$ or (salivary adj2 gland$) or Nasopharyn$ or oropharynx$ or Throat$ or oropharyng$ or hypopharynx$ or laryn$ or trachea$ or glotti$ or esophageal$ or facial$ or eyelid$ or Otorhinolary$ or ear$ or nose$ or pharyng$ or parathyroid$ or thyroid$ or papillary$) adj3 (neoplasm$ or cancer$ or tumo?r$ or malignan$ or oncolog$ or carcinom$ or $sarcom$ or metastas$ or nodule$)).tw. |
| 7 | ((head NEAR neck and surg*) or (head NEAR neck and operat*) or (neck and dissect*) or laryngectom* or pharyngectom* or HNSCC or SCCHN).tw |
| 8 | (#1 or #2 or #3 or #4) AND (#5 or #6 or #7) |
| 9 | mh diet therapy |
| 10 | (medic$ nutrition$ therap$ or cachexia$ or malnutrition$ or sarcopenia$ or clinical$ nutrition$ therap$ or medic$ diet$ therap$ or therapeutic$ diet$ or nutritional$ care$ or nutrition$ assessment$ or functional$ food$ or immunological$ diet or immunological$ nutrition$ or nutraceutical$).tw. |
| 11 | (#9 or #10) |
| 12 | (#8 and #11) |
| 13 | [mh neoplasms] or neoplasm* or cancer? or tum*r? or malignan* or oncolog* or carcinom* or leuk*mia or lymphoma? or sarcoma? |
| 14 | (#12 and #13) |

Vocabulary in Cochrane
