# Supplementary material for: Impact of nutrition counseling on nutrition status in patients with head and neck cancer undergoing radio- or radiochemotherapy: a systematic review
Source: Eur Arch Otorhinolaryngol. 2024 Jan 4;281(5):2195–209. doi: 10.1007/s00405-023-08375-1 (PMC11023997; doi:10.1007/s00405-023-08375-1)
Supplement: Supplementary file 2 — Supplementary file2 (DOCX 27 KB) [file 405_2023_8375_MOESM2_ESM.docx]

Records identified through database searching
(n = 2565 )

Screening

Included

Eligibility

Identification

Additional records identified through other sources
(n = 0 )

Records after duplicates removed
(n = 2127 )

Records screened
(n = 2127 )

Records excluded
(n = 1955 )

Full-text articles assessed for eligibility
(n = 172 )

Full-text articles excluded, with reasons
(n = 166 )

Studies included in qualitative synthesis
(n = 6 )

Studies included in quantitative synthesis (meta-analysis)
(n = 0 )
